# Daily Isoflurane Exposure Increases Barbiturate Insensitivity in Medullary Respiratory and Cortical Neurons via Expression of ε-Subunit Containing GABA _A_Rs

**DOI:** 10.1371/journal.pone.0119351

**Published:** 2015-03-06

**Authors:** Keith B. Hengen, Nathan R. Nelson, Kyle M. Stang, Stephen M. Johnson, Stephanie M. Smith, Jyoti J. Watters, Gordon S. Mitchell, Mary Behan

**Affiliations:** 1 Neuroscience Training Program, University of Wisconsin, Madison, Madison, Wisconsin, United States of America; 2 Department of Comparative Biosciences, School of Veterinary Medicine, University of Wisconsin, Madison, Madison, Wisconsin, United States of America; Kent State University, UNITED STATES

## Abstract

The parameters governing GABA_A_ receptor subtype expression patterns are not well understood, although significant shifts in subunit expression may support key physiological events. For example, the respiratory control network in pregnant rats becomes relatively insensitive to barbiturates due to increased expression of ε-subunit-containing GABA_A_Rs in the ventral respiratory column. We hypothesized that this plasticity may be a compensatory response to a chronic increase in inhibitory tone caused by increased central neurosteroid levels. Thus, we tested whether increased inhibitory tone was sufficient to induce ε-subunit upregulation on respiratory and cortical neurons in adult rats. Chronic intermittent increases in inhibitory tone in male and female rats was induced via daily 5-min exposures to 3% isoflurane. After 7d of treatment, phrenic burst frequency was less sensitive to barbiturate in isoflurane-treated male and female rats *in vivo*. Neurons in the ventral respiratory group and cortex were less sensitive to pentobarbital *in vitro* following 7d and 30d of intermittent isoflurane-exposure in both male and female rats. The pentobarbital insensitivity in 7d isoflurane-treated rats was reversible after another 7d. We hypothesize that increased inhibitory tone in the respiratory control network and cortex causes a compensatory increase in ε-subunit-containing GABA_A_Rs.

## Introduction

One of the features defining functional and regional subgroups of neurons in the CNS is the local expression of different patterns of GABA_A_R subunits [1]. Tonic GABA_A_Rs, which regulate network excitability, are principal targets of allosteric positive GABA_A_R modulators [2,3], which include ethanol [4], many anesthetics [5,6], some drugs of abuse such as barbiturates [7], and neurosteroids that are increased during pregnancy [8,9]. The expression patterns of these GABA_A_R subtypes are regulated in a compensatory manner (for review, see [10]), such that increases in allosteric modulators downregulate hippocampal and cerebellar GABA_A_Rs involved in tonic current generation [11,12]. Disruption in GABA_A_R regulation is associated with a variety of affective disorders (for review, see [13,14]), and during pregnancy, hippocampal networks are less stable and more easily rendered epileptic than in non-pregnant animals [15].

Regulation of GABA_A_R subunit expression in the CNS is complex and poorly understood, especially during pregnancy when both increases [16] and decreases [15] in hippocampal δ-subunit expression are observed. We previously described a compensatory plasticity during pregnancy in which ε subunit-containing GABA_A_Rs, which conduct a tonic current and are insensitive to many allosteric modulators [17,18, 19], are upregulated on respiratory rhythm-generating medullary neurons [20]. We hypothesized that a subunit-specific form of GABA_A_R plasticity promotes stable respiratory output by decreasing neuronal sensitivity to circulating inhibitory neurosteroids. Despite recent interest in the complex patterns of GABA_A_R subunit plasticity, it remains unclear what stimuli are required to engage these mechanisms. One possibility is that neurosteroid receptor activation, which is a potent activator of gene transcription [21], may result in a transcriptional feedback control over GABA_A_R subunit composition. Alternatively, neurosteroids can activate the PKC-dependent phosphorylation of residues on specific subunits leading to increased membrane insertion of receptor complexes [22]. Finally, neurosteroids may not be required at all. Chronic changes in activity are sufficient to induce homeostatic regulation of neuronal activity *in vivo* [23, 24], and tonic GABA_A_Rs can be recruited by cortical neurons to stabilize perturbations in channel expression [25].

While seeking an answer to these questions, we serendipitously observed that the respiratory plasticity observed during pregnancy can be induced in virgin animals: female rats exposed daily to a brief dose of isoflurane (for estrous cycle tracking) developed a phenotype strikingly similar to that of pregnant animals. Combined with data suggesting that chronic ethanol administration and pregnancy have similar effects on cerebellar and hippocampal GABA_A_Rs [15, 26], we hypothesized that GABA_A_R plasticity is stimulated in the respiratory system via repetitive manipulation of inhibitory tone. It is important to note that isoflurane acts on a variety of systems. In the nucleus ambiguus, which is adjacent to the medullary respiratory regions investigated here, isoflurane potentiates both tonic and phasic GABA_A_R inhibition [27]. While a primary target of isoflurane in medullary and spinal neurons is tonic/phasic GABAergic inhibition, others targets include glycine receptors [28] as well as excitatory synaptic currents [29].

Previously [20], we reported that ε subunit-containing GABA_A_Rs may be under activity-dependent transcriptional control because the 5’ flanking region of the gene encoding the ε subunit has conserved binding sites for CREB and SRF which are both implicated in activity-dependent neuronal gene expression and plasticity [30, 31, 32]. Similarly, GABA_A_R subunit expression patterns are partly regulated by activity dependent transcriptional control in cortical cultures [33]. Thus, we predicted that respiratory rhythm-generating neurons would increase expression of ε subunit-containing GABA_A_Rs in a predictable, compensatory manner when challenged with isoflurane.

Accordingly, we employed a chronic intermittent anesthetic exposure paradigm to determine whether increased ε subunit expression: 1) can be experimentally induced in the respiratory control network and decrease pentobarbital sensitivity both *in vivo* and in medullary slices *in vitro*, 2) can be induced in non-respiratory neurons, such as cortical neurons, 3) is restricted to only female animals. Here we demonstrate that 7d isoflurane exposures reversibly increase GABA_A_R ε subunit expression on medullary respiratory neurons, especially neurons in the PreBötzinger Complex (preBötC;) which is hypothesized to be the inspiratory rhythm generator in the mammalian brain [34, 35]. Furthermore, we show that after 30 days of treatment, GABA_A_R ε subunit expression is also increased on cortical neurons.

## Methods

### Ethical Approval

All experimental procedures were conducted in accordance with NIH guidelines and approved by the University of Wisconsin-Madison Institutional Animal Care and Use Committee (protocol V00936). A total of 95 rats (51 females and 44 males) were used in this study. All efforts were made to minimize discomfort and suffering of animals. For sacrifice, animals were either deeply anesthetized prior to decapitation.

### Isoflurane Treatment

Rats were exposed to 3% isoflurane (balance O_2_) for 5 min per day for 7d or 30d. Breathing frequency unambiguously decreased during the 5-min isoflurane exposures, although this was not systematically quantified. One group of rats was allowed to recover for 7d following a 7d treatment. Control rats were exposed to 5 min of 100% O_2_, the vehicle, or room air in the anesthetic chamber. No differences were observed between these two control treatments, thus the data were merged.

### *In Vivo* Phrenic Nerve Recordings

Three groups of rats (Sprague Dawley, Harlan) were studied: adult male and female untreated control rats (3–4 mo; n = 12), 30d isoflurane-treated female rats (3–4 mo, n = 4), and 7d isoflurane-treated male and female rats (4 mo n = 8). The methods for phrenic nerve recordings and pentobarbital dose response were described previously [20]. Briefly, rats were anesthetized initially with isoflurane (3.0–3.5%, 50% O_2_) for approximately 1 h, and then slowly converted to urethane anesthesia (1.6 mg/kg, i.v.). Rats were paralyzed (pancuronium bromide, 2.5 mg/kg. i.v.), bilaterally vagotomized and ventilated with a rodent respirator (Small Animal Ventilator, Model 683, Harvard Apparatus Inc., Holliston, MA, USA). Blood samples (60 μl) were drawn to determine arterial blood gases (PaO_2_ and PaCO_2_), pH and base excess (ABL 810, Radiometer, Copenhagen, Denmark). Body temperature was maintained at approximately 37°C using a heated table. End-tidal CO_2_ was measured with a flow-through capnograph (Capnogard, Novametrix, Wallingford, CT, USA). The right phrenic nerve was isolated via a dorsal approach, cut distally, desheathed, submerged in mineral oil and placed on bipolar, silver wire electrodes. Nerve activity was amplified (10,000x), band pass filtered (100 Hz to 10 kHz) (Model 1700, A-M Systems, Inc., Carlsborg, WA, USA) and integrated (time constant = 50 ms, Model MA-821RSP, CWE Inc., Ardmore, PA, USA).

Recordings began approximately 60 min post-surgery. The nerve was allowed to stabilize under baseline conditions of hyperoxia (PaO_2_ = 150 mmHg) and hypercapnia (PaCO_2_ = 60 mmHg). Hypercapnia was maintained throughout an experiment to ensure a chemical drive to breathe. Ten pentobarbital injections were administered (10 mg/kg/injection i.v.), each separated by 5 min. Following the final pentobarbital injection, 5 min of hypercapnic hypoxia was administered (PET CO_2_ = 80 mmHg, 13% inspired O_2_) to estimate the scope of phrenic nerve activity.

### *In Vitro* Recordings

A total of 54 rats were used for *in vitro* electrophysiology studies. Ten groups of rats were studied: adult males (3–4 months; n = 6), adult virgin females (3–4 months; n = 6), adult male and female oxygen controls (3–4 months; n = 4), adult male and female time controls (3–4 months; n = 5), adult male and female bicuculline controls (3–4 months; n = 4), 7d isoflurane-treated adult males (3–4 months, n = 6), 7d isoflurane-treated adult females (3–4 months, n = 6), 30d isoflurane-treated adult males (4 months, n = 4), 30d isoflurane-treated adult females (4 months, n = 6), 7d isoflurane-treated, 7d recovery adult males and females (3–4 months, n = 7).

Methods for *in vitro* multielectrode array recordings and analysis were described previously [20]. Briefly, brains were removed and coronal medullary and cortical slices were cut in cold (0°C) 3 mM KCl artificial cerebrospinal fluid (aCSF) with a vibrating microtome (Campden Instruments, Layfayette, IN, USA). The aCSF composition was (in mM): 120 NaCl, 26 NaHCO_3_, 20 glucose, 2 MgSO_4_, 1.0 CaCl_2_, 1.25 Na2HPO_4_, 7 KCl. Cortical slices (375 μm thick) contained primary motor and primary somatosensory areas. To remove the medulla, transverse cuts were made at caudally at spinal segment C1 and rostrally at the pontomedullary junction. A series of slices (375 μm thick) were made through the medulla from the pontomedullary junction to the obex. The first slice used for recording contained the rostral VRC [36]. The next two adjacent medullary slices used for recording contained the preBötC as identified using tissue landmarks (*i.e.*, hypoglossal nuclei were separated at the midline and the caudal extremity of the subcompact nucleus ambiguus was visible). Slices were immediately placed into an interface recording chamber (Warner Instruments, Hamden, CT, USA) and subfused with aCSF (37°C) at a rate of 8 ml/min. Slices were maintained at 37°C by an automated temperature controller (Harvard Apparatus, Holliston, MA, USA). Three 16-channel extracellular electrodes arrays (model a4x4-3μM100–177, Neuronexus, Ann Arbor, MI, USA) were placed ventrolateral to the subcompact nucleus ambiguus in VRC slices. Arrays were inserted into medullary slices at a 45° angle such that the top of the array touched the ventral border of the subcompact nucleus ambiguus. The array spanned the entire VRC and extended into tissue immediately adjacent. One array was inserted perpendicular to the cortical layers, centered on layer 3. Using this approach, multiple neurons (up to 25 neurons) were recorded from each of the three medullary slices and the cortical slice obtained from each animal in each condition. Slices were allowed to equilibrate in 7 μM KCl aCSF at 37°C with electrodes inserted for 60 min prior to recording. The following drugs were applied in our experiments: 300 μM pentobarbital (barbiturate, Fort Dodge Animal Health, Fort Dodge, Iowa, USA), 100 μM bicuculline (GABA_A_R antagonist, Tocris Bioscience, Ellisville, MO, USA), and 20 μM muscimol (GABA_A_R agonist, Tocris).

### Colocalization Immunohistochemistry

The methods for immunofluorescence and image acquisition were described previously [20]. Brain slices (375 μm) used for *in vitro* recordings were immersion-fixed in 4.0% formaldehyde, cryoprotected with a 30% sucrose solution and sectioned coronally (30 μm). Sections were first placed in blocking solution for 1h (10% NDS in 0.01 M PBS) and then reacted with antibodies against NK1-R (1:30,000; S8305; Sigma-Aldrich, St Louis, MO, USA) and GABA_A_ R ε subunit (1:1000; Abcam, Cambridge, MA, USA) applied overnight at room temperature in blocking solution and 0.3% Triton X-100. Sections were exposed to a fluorescent secondary antibody (1:300; Invitrogen, Carlsbad, CA, USA), mounted and coverslipped. Images of the entire slice were acquired at 4x and 10x. Images of the medullary ventrolateral quadrant were acquired bilaterally at 40x. There were no labeled cells in negative control slices from all conditions. A tyramine signal amplification kit (Perkin Elmer, Waltham, MA, USA) was used to diminish false-positive label between antibodies. Sections were treated according to manufacturer’s recommendations. A tyramine amplified primary antibody against NK1-R (1:30,000; Sigma-Aldrich, S8305) was applied. A biotin-SP-conjugated secondary was applied (1:200; Jackson Immunoresearch, West Grove, PA, USA) prior to the tyramine reagent (Perkin Elmer, Waltham, MA, USA). Sections were then washed and blocked again, before the application of a primary antibody against GABA_A_R ε subunit as described above (1:1000; Abcam 35971, produced against a synthetic peptide immunogen corresponding to human GABA_A_R ε subunit intracellular amino acids 237–286). Negative controls included sections reacted without primary antibodies, sections reacted without secondary antibodies, and sections reacted without tyramine signal amplification of the NK1-R primary antibody. Negative controls demonstrated specificity of antibody labeling. There were no labeled cells in control sections lacking primary antibodies.

### Image Acquisition and Analysis

Images were acquired during the same session using an Olympus Fluoview 500 laser-scanning confocal system (Tokyo, Japan) mounted on an AX-70 upright microscope. Images were analyzed using Image J software (W. Rasband, National Institutes of Health, Bethesda, MD, USA). Images were scanned with different wavelengths sequentially to prevent bleed-through across channels. User-defined thresholds were applied uniformly to all images to measure the average pixel intensity, the number of particles, and the area of particles. Total and average fluorescence was measured on cell-by-cell basis. Total intensity was calculated as the mean cell intensity times the number of cells. All positive cells more than 200 μm from the ventral edge of the slice were quantified. Background fluorescence of negative control sections was subtracted from positive label. Data were normalized to immunofluorescence levels in virgin female rats. For figures, images were uniformly processed in Adobe Photoshop (Adobe Systems Incorporated, San Jose, CA). Images (350x350 μm) were centered in the VRC based on anatomical landmarks (*i.e.*, the subcompact nucleus ambiguus was visible under brightfield conditions and demonstrated GABA_A_R ε subunit immunoreactivity). The viewfinder was centered ventral to the subcompact nucleus ambiguus, one third of the distance between the ventral edge of the slice and the subcompact nucleus ambiguus. Consistent microscope and laser settings were applied for the collection of each image.

### Statistical Analyses

For statistical analyses, R was used with function “lmer” in package “lme4” (R foundation for statistical computing, Vienna, Austria). Data were analyzed with a mixed effect linear model using experimental condition as a fixed effect. Overall group differences were tested with an F test. Subsequent post hoc comparisons were performed with a Wald T-test. Differences were considered significant if p<0.05. Data are reported as means +/− S.E.M. Categorical data (hypercapnic hypoxic challenge) were analyzed with a Pearson’s Chi-Square test (R).

### Quantitative Polymerase Chain Reaction (qPCR)

A total of 17 rats were used for qPCR experiments, including 30d isoflurane- treated rats (n = 5, 3 male, 2 female) and untreated rats (n = 12, 6 male and 6 female). Cortical and whole medullary samples contained the regions described in *in vitro* recording methods. Tissue was homogenized in Tri- Reagent (Sigma-Aldrich, St. Louis, MO, USA), and total RNA was collected according to the manufacturer’s protocol. Reverse transcription PCR (RT-PCR) was conducted using 1 μg of total RNA as a template for the reverse transcription reaction with a combination of oligo dT and random hexamer primers and ImProm-II Reverse Transcriptase (Promega, Madison, WI, USA). Quantitative RT-PCR was conducted by monitoring increases in fluorescence of SYBR-GREEN dye in real-time using the TaqMan 7300 Sequence Detection System (Applied Biosystems, Carlsbad, CA, USA). Using the comparative delta delta CT method, the relative quantities of each gene transcript were measured [37]. CT values were normalized to the levels of 18s in each sample. The following primer sequences (*rattus norvegicus*) were used in this study:

18s F: 59 AAC GAG ACT CTC GGC ATG CTA A 39

18s R: 59 CCG GAC ATC TAA GGG CAT CA 39

Epsilon F: 59 TGG AGC CTC AGC CTA GTG GAA AGA 39

Epsilon R: 59 GGC GCA GTT TAT GGT CGT AGT TGC 39

After the final amplification cycle, a dissociation curve was generated to ensure that a single gene product was amplified. Delta CT values were used for statistical analyses. Data were analyzed in R with a linear model with experimental condition as a fixed effect. For analysis of variance, function “lme” in package “nlme” was used (R foundation for statistical computing, Vienna, Austria). Post hoc analyses were conducted with a Tukey’s HSD test. Data are graphed as fold-change relative to respective untreated regional controls.

## Results

### Isoflurane Treatment Increased Resistance to Pentobarbital-Dependent Frequency Depression

To test whether chronic intermittent exposure to isoflurane was sufficient to induce a compensatory increase in ε subunit-containing GABA_A_Rs in respiratory neurons, rats were pre-treated with 5 min of 3% isoflurane per day for 7 or 30 days before testing the effects of pentobarbital on respiratory motor output. Four groups of rats were used to examine changes in phrenic nerve sensitivity to pentobarbital: 1) untreated male and female rats, 2) male rats treated for 7d with intermittent isoflurane, 3) virgin female rats treated for 7d with intermittent isoflurane, and 4) virgin female rats treated for 30d with intermittent isoflurane. Phrenic nerve burst insensitivity to pentobarbital was maximally elicited at 7d in both males and females and showed no statistical differences between 7d and 30d treatments, therefore we grouped phrenic nerve recordings into treated and untreated categories for statistical comparisons.

**Fig 1:**
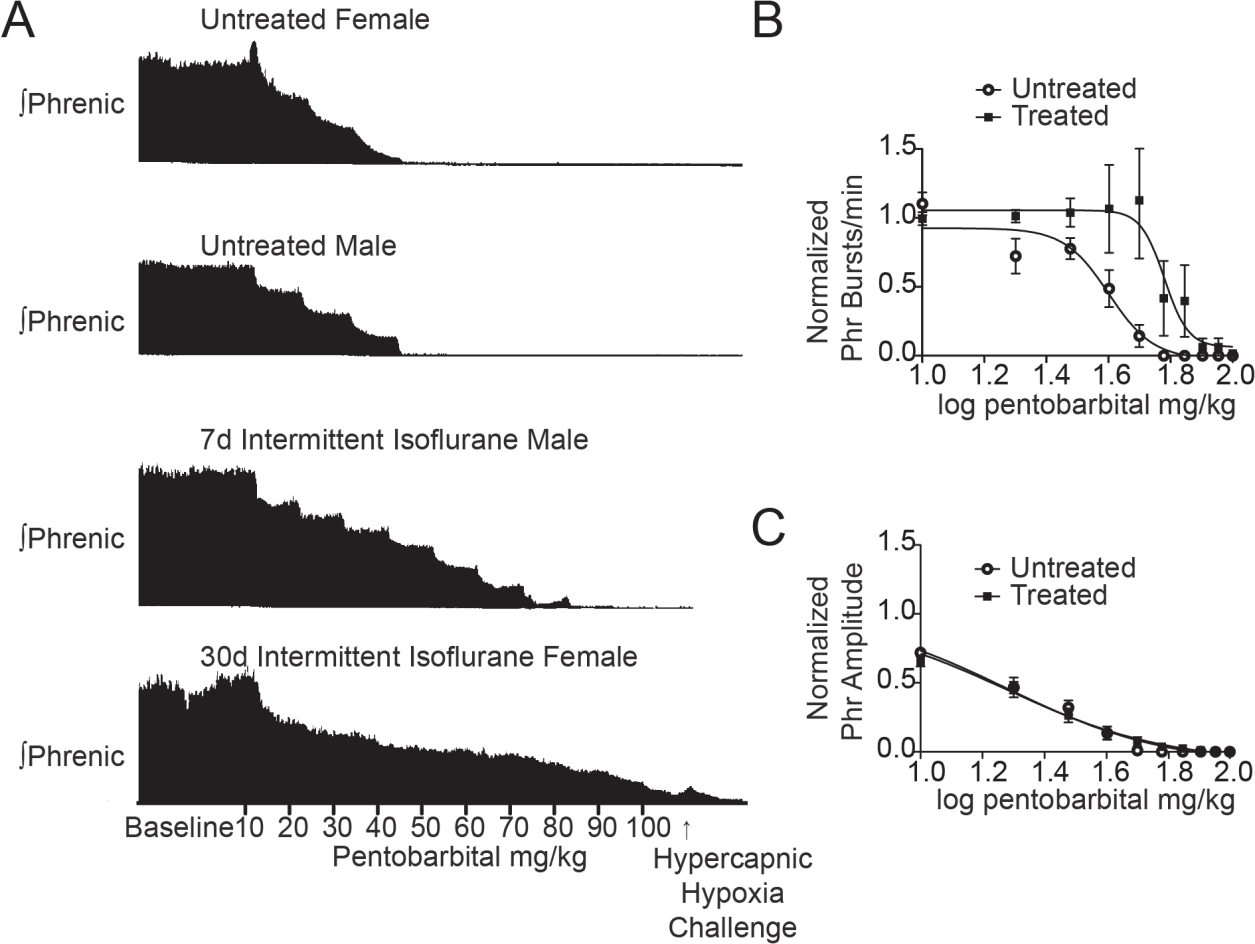
Phrenic nerve motor burst resistance to pentobarbital injections in isoflurane-treated rats. *A*, Representative phrenic neurograms from an untreated female rat, untreated male rat, 7d isoflurane-treated male rat, and 30d isoflurane-treated female rat. After establishing baseline conditions, 10 mg/kg of pentobarbital was administered every 5 min up to a maximum total dose of 100 mg/kg. The protocol ended with 5 min of hypercapnic-hypoxia (PET CO_2_ = 80 mmHg, 13% inspired O_2_) to assess phrenic nerve response to maximal chemosensory input under 100 mg/kg of pentobarbital. The upper two panels show pentobarbital-mediated inhibition of phrenic nerve bursts in untreated female and male rats. The bottom two tracings demonstrate significant resistance of phrenic nerve bursts to pentobarbital in a 7d isoflurane-treated male rat and a 30d isoflurane-treated female rat. ***C***, Pentobarbital IC50 curve of phrenic nerve burst frequency demonstrates a significant rightward shift after isoflurane treatment compared untreated rats. ***D***, Pentobarbital IC50 curve of phrenic nerve amplitude demonstrates no change following isoflurane treatment.

A dose response analysis to pentobarbital was performed using untreated (n = 6 male and 6 female) and treated (n = 4 male and 8 female) rats (Fig. 1). For untreated control male and female rats, phrenic burst frequency and amplitude decreased during sequential pentobarbital injections (top two traces in Fig. 1A) with all control rats not producing phrenic bursts by the sixth injection (60 mg/kg administered). The Hill slope of the burst frequency IC50 curve was −6.0 ± 1.7 (Fig. 1B), and the IC50 was 39.9 mg/kg (95% CI 36.0 to 44.2 mg/kg).

Following isoflurane treatment, rats continued to produce measurable phrenic motor bursts under higher doses of pentobarbital than control animals. One animal continued to produce phrenic output at the maximal dose (100 mg/kg; lowest trace in Fig. 1A). The mean dose to silence phrenic output in isoflurane-treated rats was 53 ± 7 mg/kg versus 38 ± 2 mg/kg in untreated control rats (p<0.05). The Hill slope increased to −11.4 ± 9.1 (Fig. 1B) and the burst frequency IC50 significantly increased to 60.3 mg/kg (95% CI 51.9 to 70.0 mg/kg; p<0.01). To normalize the lethal dose of pentobarbital across rats, individual approximate Hill slopes were calculated for burst frequency and averaged for treated and untreated groups. This revealed that the slope in isoflurane-treated rats (−218.4 ± 71.1) was significantly steeper than in untreated control rats (−57.6 ± 30.4, p<0.05).

In contrast to the pentobarbital-dependent decrease in phrenic burst frequency, the amplitude of phrenic nerve output was highly variable following pentobarbital application. The parameters defining the best-fit models for the phrenic nerve amplitude dose response curve did not differ by condition (p = 0.35; Fig. 1C), thus one IC50 (19.1 mg/kg; 95% CI 17.3 to 21.1 mg/kg) and one Hill slope (−1.68 ± 0.15) described the entire amplitude dataset. These data indicate that following isoflurane treatment, pentobarbital was still inhibitory on neurons contributing to phrenic nerve amplitude but not burst frequency.

To assess whether respiratory networks were truly silenced by 100 mg/kg pentobarbital, we sought to provide maximal stimulus for breathing to prompt the system to surmount barbiturate-induced depression. There was notable variability in the dose of pentobarbital required to silence the phrenic nerve. While ≤60 mg/kg of pentobarbital was sufficient to depress phrenic nerve activity in all untreated control rats, not all isoflurane-treated rats were depressed by the maximal dose of 100 mg/kg. To assess whether the degree of inhibition differed between groups, we challenged rats with a strong stimulus (hypercapnic-hypoxia challenge) at the end of each experiment (Fig. 1A). Six of 12 isoflurane-treated rats mounted a detectable phrenic response to the challenge, while only two of 12 untreated control rats responded (p = 0.08).

### Pentobarbital Sensitivity of Spontaneous Neuronal Activity in Medulla and Cortex

During sequential pentobarbital injections, respiratory phrenic burst frequency was sustained, thereby suggesting that respiratory rhythm-generating neurons in medulla express pentobarbital-insensitive GABA_A_Rs. In addition, it is not clear whether isoflurane exposures increase the expression of pentobarbital-insensitive GABA_A_Rs in other parts of the CNS. To answer these questions, pentobarbital sensitivity was examined in the neural regions responsible for rhythm generation (VRC, preBötC) as well as in cortical neurons, which exhibit a GABA_A_R mediated tonic current [38]. Four groups of rats were tested: 1) untreated male and female rats, 2) 7d isoflurane-treated male and female rats, 3) 30d isoflurane-treated male and female rats, and 4) male and female rats that were allowed 7d of recovery following 7d isoflurane treatment (data from the untreated groups are reported in [20]).

**Fig 2:**
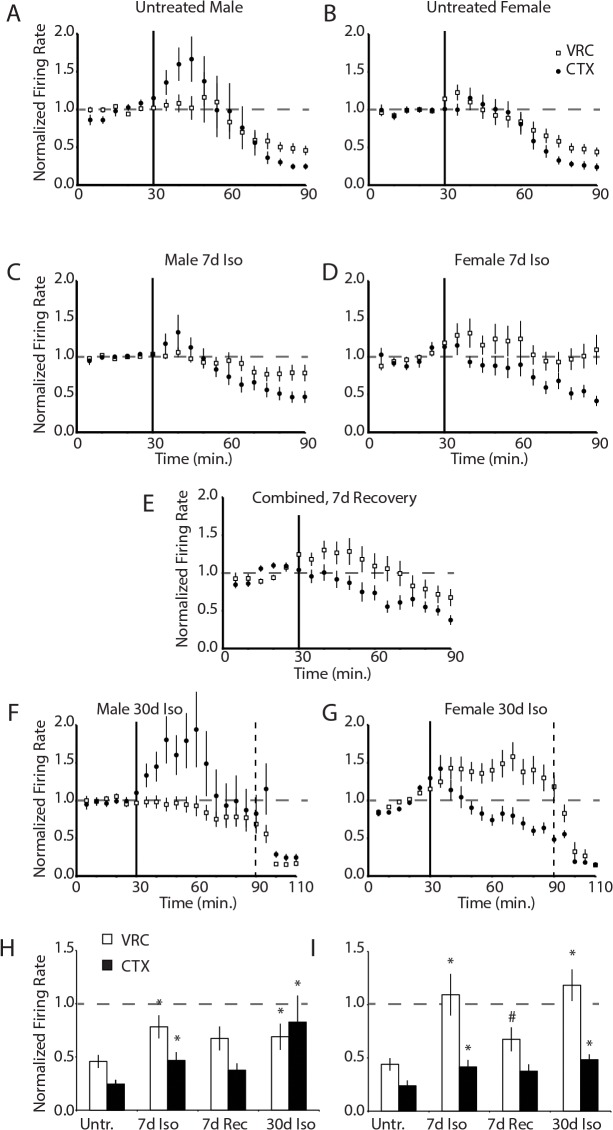
Spontaneous neuronal activity resistance to bath-applied pentobarbital in medullary and cortical slices from 7d isoflurane-treated rats. Spontaneous activity of neurons in the region anatomically consistent with the VRC and PreBötC (all labeled as VRC neurons) are insensitive to bath-applied pentobarbital (300 μM) following 7d isoflurane treatment. ***A***, Mean normalized firing rate of VRC (n = 62, open circles) and CTX (n = 37, filled circles) neurons from male rats in response to 300 μM pentobarbital (applied at vertical bar, t = 30 min). ***B***, Pentobarbital had equivalent depressive effects on VRC (n = 37) and CTX (n = 47) neurons from virgin female rats. ***C***, Following pentobarbital treatment, spontaneous activity of VRC neurons (n = 62) from male rats treated with intermittent isoflurane for 7d were significantly more active than VRC neurons from untreated control rats. The response of cortical neurons (n = 49) to pentobarbital was non-significantly shifted in the same direction. ***D***, Likewise, in female rats treated with isoflurane for 7d, pentobarbital failed to inhibit spontaneous neuronal activity in the VRC neurons (n = 40). CTX neurons (n = 51) trended non-significantly towards an increase in activity compared to untreated control female rats. ***E***, This effect was reversible, as 7d of recovery following 7d of intermittent isoflurane shifted spontaneous activity of VRC (n = 27) and CTX neurons (n = 38) in rats toward baseline values. ***F***., 30d of intermittent isoflurane significantly attenuated the pentobarbital response in VRC (n = 40) and CTX neurons (n = 32) in male rats. To confirm the presence of pentobarbital-insensitive GABA_A_Rs, muscimol (20 μM) was applied at 90 min (dashed vertical line). Nearly all neurons that were resistant to pentobarbital were inhibited by muscimol. ***G***, In female rats exposed to 30d isoflurane treatment, the response of VRC (n = 123) and CTX (n = 83) neurons to pentobarbital was significantly attenuated. These neurons were also silenced by muscimol (dashed vertical line). (***H*,*I*)** The average steady-state responses to bath-applied pentobarbital are quantified for the different isoflurane treatments and recovery for male rats (***C***) and female rats (***D***). The asterisk indicates p<0.05 for comparison to control while the pound sign indicates p<0.05 for comparison to 7d treatment.

Consistent with prior data [39, 40, 20], in slices from untreated control male (n = 6) and female rats (n = 6), 60 min exposure to bath-applied 300 μM pentobarbital inhibited spontaneous activity in VRC neurons to 44 ± 15% of baseline (n = 103 neurons, p<0.05) and cortical (CTX) neurons to 24 ± 8% of baseline (n = 84 neurons, p<0.05) compared to time controls (n = 5 animals, 56 VRC neurons and 48 CTX neurons, 136 ± 19% and 123 ± 19% of baseline, respectively) (Fig. 2A, B). There were no significant differences between untreated male and female animals.

The 7d intermittent isoflurane treatment (n = 12 rats) reduced the inhibitory effects of 1h pentobarbital on VRC (n = 102 neurons, 91 ± 21% of baseline, p<0.05) and CTX neurons (n = 84 neurons, 49 ± 10% of baseline, p<0.005) compared to untreated control rats (Fig. 2C, D). There were no significant differences between male and female animals. The effects of 7d isoflurane treatment were reversible in the VRC, as 7d recovery significantly shifted the effect of 1h pentobarbital towards the response of untreated control rats (VRC, n = 27 neurons, 44 ± 14% of baseline, p<0.01) (Fig. 2E). There was no significant difference between spontaneous activity after pentobarbital application in CTX neurons from 7d isoflurane-treated rats and 7d recovery rats.

The 30d of intermittent isoflurane treatment rendered VRC neurons insensitive to pentobarbital application (n = 163 neurons, 104% of baseline, p>0.05 compared to time control) (Fig. 2F, G). The 30d intermittent isoflurane treatment differentially affected CTX neurons in males and females (p<0.05). In males, CTX neurons maintained 83 ± 18% of baseline activity (n = 33 neurons) (Fig. 2F) while in females, CTX neurons maintained 48 ± 9% of baseline activity (Fig. 2G). In both males and females, CTX neurons were significantly less inhibited by pentobarbital after 30d of intermittent isoflurane than untreated animals (female, p<0.05; male, p<0.005).

The persistence of spontaneous neuronal activity following pentobarbital application to the CTX and VRC of 30d treated animals could reflect either a lack of GABA_A_Rs on neurons, or the presence of pentobarbital-insensitive GABA_A_Rs. To address this question, 20 μM muscimol (GABA_A_R agonist) was bath-applied to from slices from isoflurane-treated rats after 1h of 300 μM pentobarbital (Fig. 2F, G). After 1h of exposure to 300 μM pentobarbital, CTX and VRC neurons from isoflurane-treated rats were rapidly (<5 min) inhibited by muscimol, confirming the presence of functional, pentobarbital-insensitive GABA_A_Rs. The response of CTX and VRC neurons to muscimol did not differ between isoflurane-treated rats and time control rats (p>0.05), indicating the presence of pentobarbital-insensitive GABA_A_Rs in CTX and VRC after 30d of isoflurane.

The inhibitory effects of pentobarbital in CTX and VRC neurons were mediated by GABA_A_Rs, as bath application of 100 μM bicuculline (GABA_A_R antagonist) to slices (n = 4 rats; 2 male, 2 female) prior to co-application of 100 μM bicuculline and 300 μM pentobarbital prevented the inhibition of spontaneous activity of both CTX and VRC neurons by pentobarbital observed in virgin female and male slices (data not shown).

A summary of the steady-state pentobarbital responses in VRC and CTX neurons in slices from male and female rats shows that isoflurane exposures increased pentobarbital resistance after 7d and 30d (Fig. 2H, 2I). The response in female rats was greater compared to male rats. Together, these data suggest that intermittent isoflurane treatment is sufficient to increase the expression of a pentobarbital-insensitive GABA_A_R subtype in VRC and CTX neurons.

### Immunohistochemical Localization of ε Subunit Expression in Medulla and Cortex

**Fig 3:**
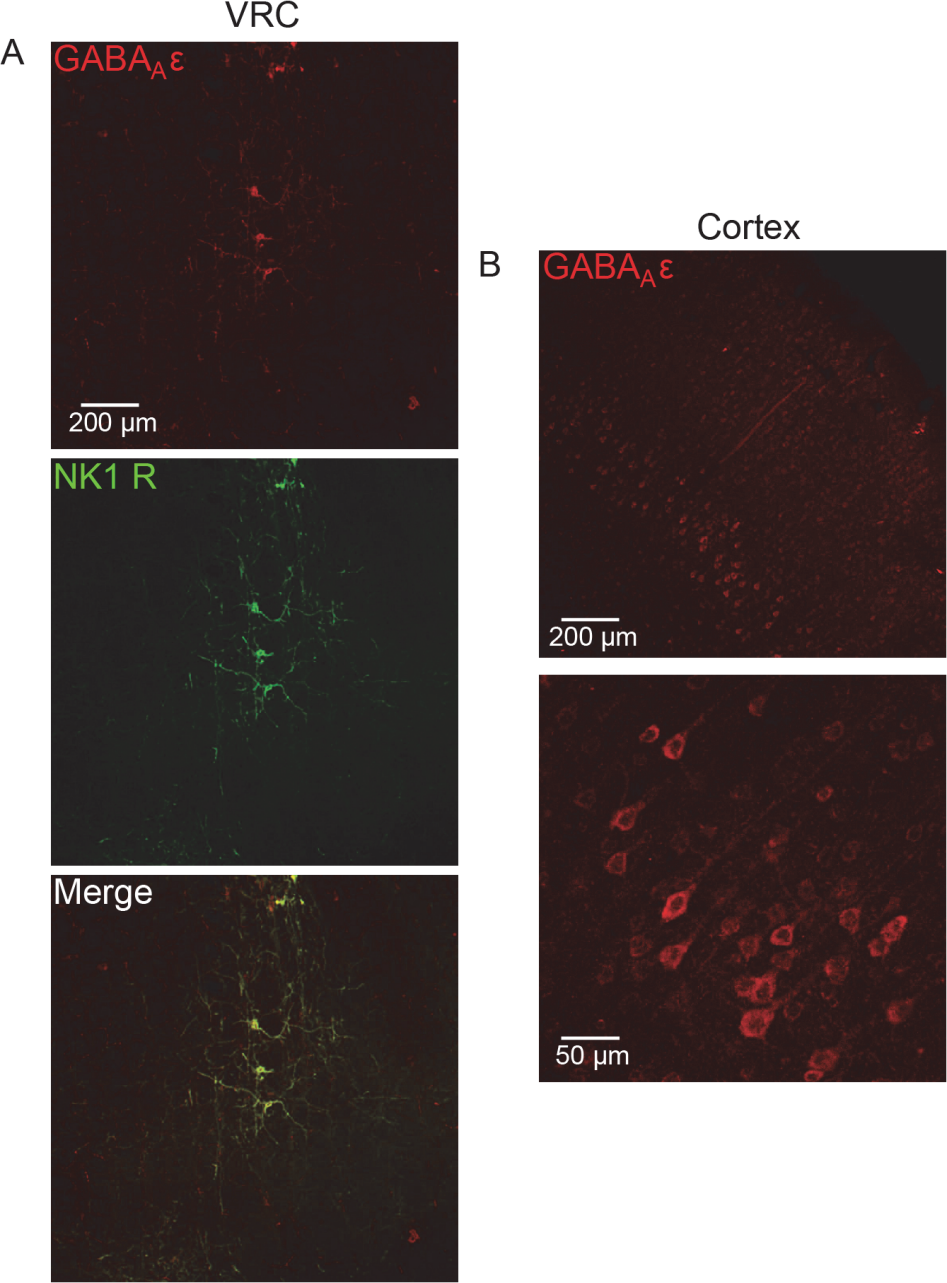
GABA_A_R ε subunit protein expression in medulla and cortex of isoflurane-treated rats. ***A***, Medullary slices used for *in vitro* electrophysiology were immunohistochemically labeled for the GABA_A_R ε subunit (red, top) and NK1-R (green, middle). Images were acquired in the location of electrode placement. The GABA_A_R ε and NK1-R exhibited >90% colocalization on neurons in the preBötC of the medulla (merge, bottom). Example image was acquired in the preBötC of a virgin female rat. ***B***, Cortical slices previously used for *in vitro* electrophysiology were sectioned and immunohistochemically labeled for the GABA_A_R ε subunit. Example image was acquired in the cortex of a 30d isoflurane-treated male rat.

To confirm the presence or absence of GABA_A_R ε subunit expression at the *in vitro* recording sites, medullary slices from recording experiments were treated with an antibody against the GABA_A_R ε subunit. Medullary sections were co-labeled for GABA_A_R ε subunit and for NK1-R (Substance P receptor) that is used as a marker for putative respiratory neurons in the VRC and preBötC [41, 42]. NK1-R and the GABA_A_R ε subunit exhibited >90% colocalization, both in the nucleus ambiguus (subcompact and compact; Lin et al., 2008), which was used as a landmark for electrode placement, and in the VRC (Fig. 3A; [36]). Neurons in the VRC displayed enriched somatic ε subunit staining as well as in primary neuronal processes (Fig. 3A). The pentobarbital-insensitive GABA_A_Rs identified *in vitro* were functionally consistent with immunohistochemically identified ε subunit-containing GABA_A_Rs in the VRC. To our surprise, the GABA_A_R ε subunit was detectable in CTX neurons in all groups as well. Layer 5 pyramidal cells demonstrated the most robust expression, and staining appeared to be largely restricted to the soma (Fig. 3B).

### ε Subunit mRNA Expression in Medulla and Cortex

**Fig 4:**
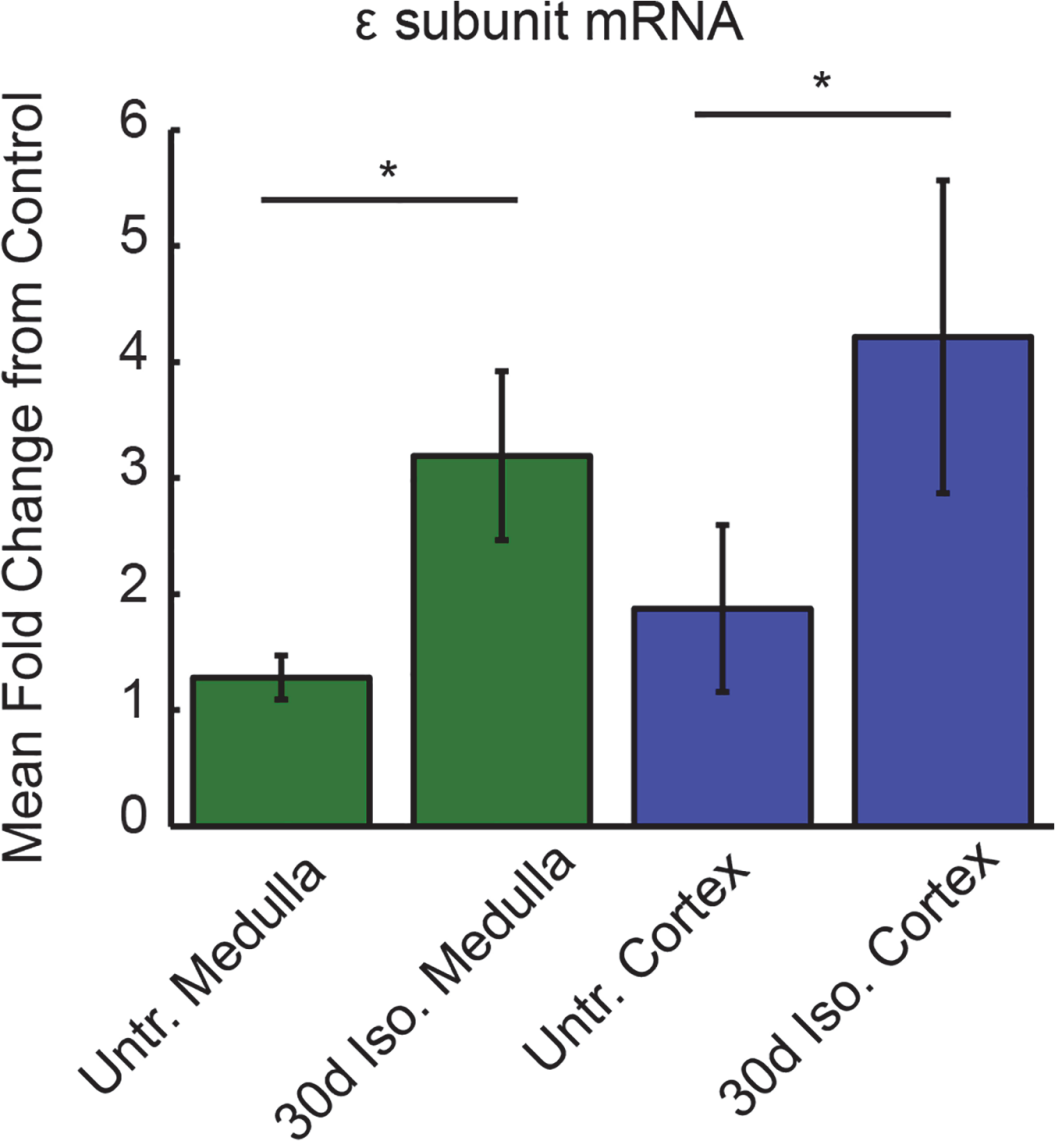
Increased GABA_A_R ε subunit mRNA levels in medulla and cortex in 30d isoflurane-treated rats. Medullary GABA_A_R ε mRNA levels were more than 3-fold greater in 30d isoflurane-treated rats compared to untreated control rats. Likewise, cortical GABA_A_R ε mRNA levels were more than 4-fold increased in 30d isoflurane-treated rats. Statistics were conducted on delta CT values. Asterisk denotes p<0.05. Error bars indicate SEM.

To quantify whether intermittent isoflurane treatment altered the transcriptional regulation of the GABA_A_R ε subunit, cortical and medullary samples from 30d isoflurane-treated rats (n = 5) and untreated control rats (n = 12) were harvested for GABA_A_R ε subunit mRNA analysis (Fig. 4). The 30d isoflurane treatment increased GABA_A_R ε subunit mRNA in medullary samples compared to untreated samples (3.9 ± 1.39-fold increase, p<0.05). Cortical samples from 30d isoflurane-treated rats also demonstrated increased GABA_A_R ε subunit mRNA levels compared to controls (4.2 ± 1.35-fold increase, p<0.05, Wilcoxon Rank Sum). These data demonstrate a correlation between the emergence of barbiturate-insensitive GABA_A_Rs in VRC and cortex and the transcriptional upregulation of GABA_A_R ε subunit mRNA.

## Discussion

Multiple forms of neuronal plasticity must be carefully coordinated to produce a stable and functional nervous system. The mechanisms that are actively expressed are regulated both developmentally and spatially, and depend upon external and internal environments. This study shows that repeated exposure to brief episodes of an inhaled anesthetic is sufficient to functionally reorganize medullary and cortical neuronal circuitry. Following 7 days of 5 min/day exposure to isoflurane, both *in vivo* and *in vitro* measures of respiratory neuronal output revealed a significantly increased barbiturate tolerance. After 30 days of treatment, cortical networks were similarly affected.

This study initially sought to test whether non-pregnant animals could be induced to increase ε subunit expression and barbiturate tolerance in VRC neurons. Our findings make it clear that gestational hormones are unnecessary for this effect, as both virgin female and male rats have the capacity to express this form of plasticity. We cannot rule out the effects of neurosteroids, as there is evidence that GABAergic modulators may increase neurosteroid levels in *in vitro* hippocampal preparations [43]. Surprisingly, our data also reveal that this phenomenon is not restricted to medullary neurons, as cortical neurons displayed a similar pharmacological profile following longer treatment periods. Whether this is a global feature of the cortex is unclear, but these data suggest that the incorporation of pharmacologically distinct GABA_A_R configurations is a general mechanism of neuronal plasticity.

If this plasticity is not gated by the interaction of hormones and nuclear receptors (one of our initial hypotheses), how might this process be triggered in neurons? Long duration (minutes to hours) application of GABA to cultured neurons leads to an increase in receptor internalization, an uncoupling of the allosteric modulatory site from the GABA binding site, and a decrease in the expression of specific subunits [33]. The mechanism linking increased allosteric modulatory pressure and the regulation of the ε subunit is unclear. This will be a critical question to answer in future studies.

The demonstration that the frequency of phrenic nerve output *in vivo* is highly resistant to sequential pentobarbital injections in isoflurane-treated rats strongly suggests that rhythm-generating neurons in the brainstem increase ε subunit expression in GABA_A_Rs. Since it is hypothesized that inspiratory activity produced mainly by preBötC neurons in the VRC [44, 34, 35], pentobarbital sensitivity of spontaneous neuronal activity was tested in medullary slices from control and isoflurane-treated rats. None of the recorded neurons in the medullary slice experiments could be defined as being respiratory-related because adult medullary slices do not produce spontaneous respiratory rhythmic motor activity similar to that produced in neonatal rodent medullary slice [44]. However, it is likely most of the recorded neurons were respiratory-related preBötC neurons because the preBötC region has distinctive surrounding neural landmarks that define the region and are easily visualized in medullary slices *in vitro*. Furthermore, ε subunit expression was increased in NK1-R-positive neurons within the preBötC region in isoflurane-treated rats. Since the NK1-R is one of several markers for preBötC neurons [45], this finding is consistent with the hypothesis that ε subunit expression increased in preBötC neurons to compensate for the isoflurane-dependent decrease in respiratory motor activity. This study does not address the question as to whether intermittent isoflurane exposures altered ε subunit expression in other respiratory-related brainstem regions controlling expiration, postinspiration, or central chemosensation. Furthermore, modulatory neurons (*e.g.*, serotonergic, noradrenergic, dopaminergic) projecting to the preBötC may also have increased expression of ε subunits in GABA_A_Rs, and thereby contributed to the altered the responsiveness of the respiratory control network to pentobarbital.

The *in vivo* and slice physiology data presented here provide measures of network activity as opposed to cell autonomous responses. Our data demonstrate the presence of pentobarbital insensitive GABA_A_Rs in the cortex and the brainstem, but it is difficult to predict specific expression patterns from these experiments. For example, the transient increases in cortical neuronal activity of 30d isoflurane treated male rats (Fig. 2F) likely represent pentobarbital-mediated inhibition of a subclass of inhibitory interneuron, resulting in disinhibition of excitatory cells. Subunit distribution rules will be important to understand in the future.

ε subunit-containing GABA_A_Rs are insensitive to most allosteric positive modulators [18, 19]; both the human and rat isoform of the ε subunit confers anesthetic insensitivity to neurons [46], and GABA_A_Rs containing the ε subunit are upregulated on VRC neurons during pregnancy when pentobarbital sensitivity decreases [20]. Our data fall short of establishing a mechanistic link between the induced expression of ε subunit-containing GABA_A_Rs and barbiturate insensitivity. We do, however, demonstrate the conditional presence of fully functional, barbiturate-insensitive GABA_A_Rs in brain regions that are immunohistologically enriched with the ε subunit and show increased ε subunit mRNA transcription following treatment. Furthermore, these receptors are rapidly inhibited by muscimol, and the inhibitory actions of pentobarbital are blocked by bicuculline (indicating the involvement of GABA_A_Rs). These data are difficult to understand without the specific pharmacological properties imbued by the ε subunit, thus this is the most likely explanation for our findings. However, because the connection between the ε subunit and the respiratory plasticity described here is correlative, it will take further investigation to unequivocally confirm a molecular mechanism.

Cortical networks have the capacity to homeostatically regulate gain control via plasticity of intrinsic excitability and changes in synaptic strength such that network activity is maintained following perturbations in drive [24, 47, 48]. Similarly, precise balancing of respiratory neuronal input/output ratio is necessary for survival, and gain modulation of respiratory rhythm generating neurons is provided by a tonic GABA_A_R current [49]. Neurosteroids, like barbiturates, act on these currents and suppress activity in medullary slices from naïve animals [50]. Thus, these data suggest a compensatory mechanism of gain modulation that counteracts the hyperpolarizing properties of chronic exposure to allosteric positive GABA_A_R modulators, such as neurosteroids, ethanol, and in some cases, anesthetics. Without a compensatory response to allosteric modulators, respiratory pathologies would arise as a result of pregnancy, regular alcohol consumption, or multiple exposures to anesthetics. To our knowledge, these are the first data to demonstrate reorganization of the neural control of respiration by repeated anesthetic exposure. Prior data demonstrate the ability of anesthetics to alter GABA_A_R expression patterns. Sekine et al. (2006) described changes in forebrain mRNA for the GABA_A_R α_4_ subunit during anesthesia with propofol or isoflurane [51]. Similar to our findings, chronic ethanol exposure is associated with a cross-tolerance to many GABAergic anesthetics [26, 52, 53]. It is likely that cross-tolerance is a feature of tonic GABA_A_R plasticity, as we have previously described VRC insensitivity to both ETOH and barbiturates in hibernating ground squirrels [40].

## Significance

This study presents a novel, clinically relevant, inducible model of neuroplasticity that was clearly demonstrated in an intact animal with measurable, functional spinal respiratory motor output. We provide evidence that this isoflurane-induced neuroplasticity likely involves the expression of a relatively unknown, yet highly conserved, GABA_A_R subunit that confers insensitivity to allosteric modulation by neurosteroids, anesthetics, ethanol, barbiturates, and benzodiazepines [18, 19, 39, 40, 20]. Here we demonstrate that this plasticity is restricted neither to specific classes of animals (i.e. pregnant or hibernating), nor respiratory neurons. These data raise the possibility that this type of respiratory neuroplasticity, which mirrors some of the changes associated with hibernation and pregnancy, is inducible and may be harnessed to treat pathological conditions.

Many long-term *in vivo* preparations in basic neurobiology research rely on regular administration of isoflurane and other GABAergic anesthetics in order to prepare an animal for data collection. The findings presented here suggest that these protocols may be inadvertently manipulating the very networks being studied. The impact of these methods needs further study.
